# Observation of Clinicopathologic Features of Pituitary Adenoma With Neuronal Differentiation

**DOI:** 10.3389/fendo.2022.848762

**Published:** 2022-03-15

**Authors:** Limei Zheng, Xiaorong Yan, Chengcong Hu, Peng Zhang, Yupeng Chen, Qiaoyan Zheng, Liwen Hu, Mi Wang, Guoping Li, Ping Wu, Changzhen Jiang, Jing Tian, Sheng Zhang, Xingfu Wang

**Affiliations:** ^1^ Department of Pathology, The First Affiliated Hospital of Fujian Medical University, Fuzhou, China; ^2^ Department of Neurosurgery, The First Affiliated Hospital of Fujian Medical University, Fuzhou, China; ^3^ Department of Cardiovascular Surgery, Renji Hospital, Shanghai Jiaotong University School of Medicine, Shanghai, China; ^4^ Department of Immunology and Microbiology, Shanghai Institute of Immunology, Shanghai Jiao Tong University School of Medicine, Shanghai, China

**Keywords:** mixed-gangliocytoma pituitary adenoma, pituitary tumor, neural differentiation, acromegaly, PIT1

## Abstract

**Objective:**

To investigate the clinicopathologic features of pituitary adenoma with neuronal differentiation.

**Methods:**

Four patients with mixed gangliocytoma-pituitary adenomas between January 2011 and January 2021 and 111 new-onset patients with adenomas between January 2019 and June 2021 who attended the First Affiliated Hospital of Fujian Medical University were included in the study. The histological and immunohistochemical findings were analyzed. Neuronal differentiation marker staining was performed on new-onset adenomas, and the related literature was reviewed.

**Results:**

Altogether, more than 100 mixed gangliocytoma-pituitary adenoma cases have been reported in the literature until now, of which pituitary-specific POU-class homeodomain transcription 1 (PIT1) positive adenomas are more frequently observed. In the present study, all 4 patients we described were female, aged 29 to 53 years (mean 39 years). Clinically, 3/4 patients presented with acromegaly, and 1/2 patients presented with headache. Histologically, the tumor was composed of two distinct mixed components. The one was a population of neoplastic ganglionic cells with large nuclei, prominent nucleoli, and abundant basophilic cytoplasm embedded in a fibrillary background. Stains of chromograninA (CgA), synaptophysin (Syn), Calretinin (CR) were positive. Axotomy-like expression was observed in neurofilament (NF) staining. PIT1 was expressed in partial ganglionic cells in all cases. The other component was a population of small uniform cells with round nuclei and acidophilic cytoplasm. Prolactin (PRL) and growth hormone (GH) were positive in all 4 cases. PIT1 was positive in the nuclei of adenomas. Although adenomas and ganglionic regions varied in histology, there was a population of cells with neuronal differentiation expressing PIT1. Additionally, axotomy-like expression of NF staining could be seen in a distant area of adenoma regions. A total of 111 cases of adenomas without ganglionic cells were included in this study, including 7 cases with neuronal differentiation. Among them, 4 cases were prolactinomas, 2 cases were somatotroph adenomas, and 1 case was corticotroph adenoma. 6/7 cases were PIT1-positive adenomas. And the remaining one case is T-PIT-positive adenoma.

**Conclusions:**

Mixed gangliocytoma-pituitary adenomas are rare tumors with neuronal differentiation. The majority of MGAs are associated with endocrinopathies, mainly acromegaly. Our results suggest that PIT1-positive pituitary adenomas may have neural differentiation potential, which may not be unusual. This indication supports the possibility that the neuronal transdifferentiation of adenomatous cells is a possible mechanism, and the underlying mechanism requires further elucidation.

## Introduction

Gangliocytomas/mixed gangliocytoma-adenomas (GCs/MGAs) are rare entities in the sellar region and are categorized as neuronal and paraneuronal tumors according to the 2017 World Health Organization Neuroendocrine Tumor Classification Guideline (1). Most cases reported are composed of ganglion cells with pituitary adenomas (2), forming so-called mixed gangliocytoma adenomas. Isolated gangliocytomas are extremely rare. In the present study, 4 cases of mixed gangliocytoma adenoma and 111 new-onset cases of pituitary adenoma were analyzed. The purpose of this study was to investigate the clinicopathologic features of pituitary adenoma with neuronal differentiation.

## Materials And Methods

We retrospectively studied the histological examinations of 4 patients with mixed gangliocytoma-pituitary adenomas between January 2011 and January 2021 and 111 new-onset patients with adenomas between January 2019 and June 2021 who attended the First Affiliated Hospital of Fujian Medical University. For histology and immunohistochemistry, the tissue was fixed in 10% formalin and subsequently paraffin embedded. Paraffin-embedded sections (4–6μm thick) were processed, and then selected blocks were stained with antibodies to transcription factors and pituitary hormones, including PIT1 (1:500, G-2; Zsbio), steroidogenic factor 1 (SF-1) (1:500, OTI1H2; Zsbio), T-box family member TBX19 (T-PIT) (1:500, OTI2G1; Zsbio), adrenocorticotropic hormone (ACTH) (RAB-0010; Maxim), PRL (MAB-0886; Maxim), follicle-stimulating hormone (FSH) (MAB-0782; Maxim), GH (MAB-0883; Maxim), luteal hormone (LH) (MAB-0788; Maxim) and thyroid stimulating hormone (TSH) (MAB-0796; Maxim). Immunohistochemistry (IHC) stains that have been utilized for the detection of neuronal structures include neuronal nuclei (NeuN) (1:200; MAB-0578; Maxim), CR (1:200; ZA-0026; Zsbio), NF (1:300; TA309765; Zsbio), Syn (1:600; ZA-0263; Zsbio) and MAP2 (1:200; ZA-0380; Zsbio). Other antibodies for diagnosis and differential diagnosis includes CK8 (MAB-1002; Maxim), thyroid transcription factor 1 (TTF-1) (HPA054837; Roche), BRAF (HPA001328; Roche), CD34 (Kit-0004; Maxim), GFAP (1:300; ZM-0118; Zsbio), P53(MAB-0674; Maxim) and Ki-67 (1:400; ZM-0378; Zsbio). Double-labeling IHC assay PIT-1/MAP2 was performed using dual detection kit (Roche) in BenchMark ULTRA system.

Medical files were retrospectively reviewed, and magnetic resonance imaging (MRI) studies of the patients were also analyzed. The follow-up information came from outpatient follow-up review or telephone follow-up.

## Results

### Case 1

A 53-year-old female presented with a 12-month history of acromegaly. She had a history of surgical thyroidectomy. MRI showed an intrasellar mass measuring 2.1 cm×1.6 cm×1.5 cm. The tumor passed the intercarotid line, but not beyond the tangent on the lateral aspects of the intracavernous. Random GH serum level was elevated at 19.48 μg/L on admission. Insulin-like growth factor-1 (IGF-1) level was 408 ng/mL (normal value 87-238 ng/mL). A 75g oral glucose tolerance test (OGTT) achieved inadequate suppression of nadir GH level (17.97ng/mL; normal value 0.06-5μg/L). Other hormones were within normal range. She underwent a transsphenoidal endoscopic approach resection of the tumor. At the 50th month follow-up after the surgery, the clinical symptoms of the patient markedly improved.

### Case 2

A 29-year-old female with acromegaly complained of a 1-year history of headache with aggravation for 1 week. MRI showed a mass measuring 2.4 cm×2.3 cm×1.8 cm in the sella turcica. On coronal contrast imaging, the intracavernous artery was totally encased by the tumor. The boundaries were relatively clear. Preoperative growth hormone level was 4.28μg/L and IGF-1 level was 516.6 ng/mL (normal value 63-373ng/mL). 75g OGTT found nadir GH level of 4.79ng/mL, resulting in no suppression of less than 1μg/L. PRL serum level was elevated at 565.4mIU/L. Other pituitary hormones were within normal range. The patient was followed up for 12 months after transsphenoidal resection, and her general condition was good.

### Case 3

**Figure 1:**
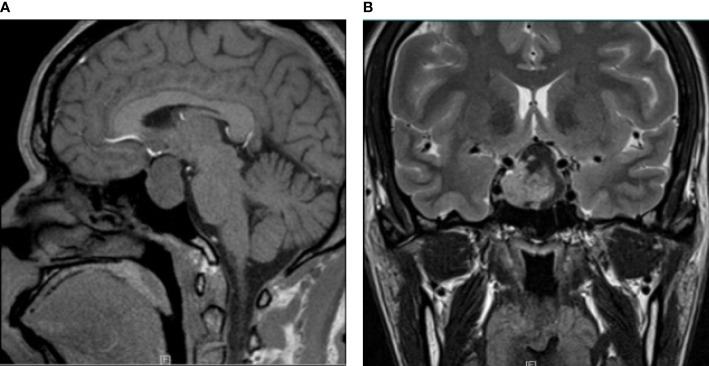
Radiological image of mixed gangliocytoma-pituitary adenoma. **(A)** Preoperative MRI shows a lesion with sagittal hypointensity on T1-weighted imaging. **(B)** T2-weighted coronal contrast imaging shows a hyperintensity signal with a prominent waist sign. The tumor passed the tangent of the medial aspects of the intracavernous and supracavernous internal carotid arteries, but did not extend beyond the intercarotid line.

A 35-year-old female had a 5-year history of secondary amenorrhea and a 1-year history of acromegaly. MRI revealed an intra- and suprasellar lesion with a prominent waist sign (**Figure 1**) measuring 3.0 cm×2.7 cm×2.0 cm. The tumor passed the medial tangent, but did not extend beyond the intercarotid line. In addition, it was heterogeneous enhanced after contrast. Laboratory tests revealed high levels of GH (70.10μg/L), PRL (1160.0mIU/L) and IGF-1 (545.2ng/mL, normal value 63-373ng/mL). Other hormones were within normal range. Transsphenoidal endoscopic gross total tumor resection was performed. At a follow-up visit 7 months after surgery, all clinical symptoms had disappeared. Menstruation was restored following surgery but irregular.

### Case 4

A 39-year-old female was admitted to the hospital with severe headache for ten days. MRI showed an intrasellar equal T1 and slightly long T2 signal, within the intercarotid line. The size of the lesion was 1.8 cm×1.5 cm×1.4 cm. GH serum level was elevated at 16.55μg/L and IGF-1 level was elevated at 640 ng/mL (normal value 63-373ng/mL). Serum PRL level was elevated at 437.9mIU/L. Other hormones were within normal range. The mass was excised *via* an endoscopic transsphenoidal procedure. The postoperative record was taken at 4 months after surgery, and the clinical symptoms improved significantly.

The patients’ clinical characteristics are summarized in **Table 1**.

**Table 1:** List of clinical characteristics of the patients.

	Case1	Case2	Case3	Case4
Gender	F	F	F	F
Age (year)	53	29	35	39
Location	intrasellar	Intrasellar, suprasellar, cavernous sinus	Intrasellar, suprasellar	intrasellar
Clinical presentation	Acromegaly progressing	Headache, acromegaly	Acromegaly, amenorrhea	Headache
Tumor size (cm)	2.07×1.58×1.52	2.4×2.3×1.8	3.0×2.7×2.0	1.8×1.5×1.4
Knosp grade	II	IV	I	I
GH level (μg/L)	19.48	4.28	70.10	16.55
nadir GH level after OGTT	17.97	4.79	/	/
IGF-1 level(ng/mL)	408	516.6	545.2	640
PRL level(mIU/L)	53.2	565.4	1160.0	437.9
Surgery	ETS	ETS	ETS	ETS
Follow-up (month)	50, NED	12, NED	7, NED	4, NED

F, Female; ETS, endoscopic transsphenoidal surgery; NED, No evidence of disease.

Normal ranges: GH: 0.06-5μg/L, IGF-1:63-373ng/mL (Case1: 87-238 ng/mL), PRL: 86-324 mIU/L.

**Figure 2:**
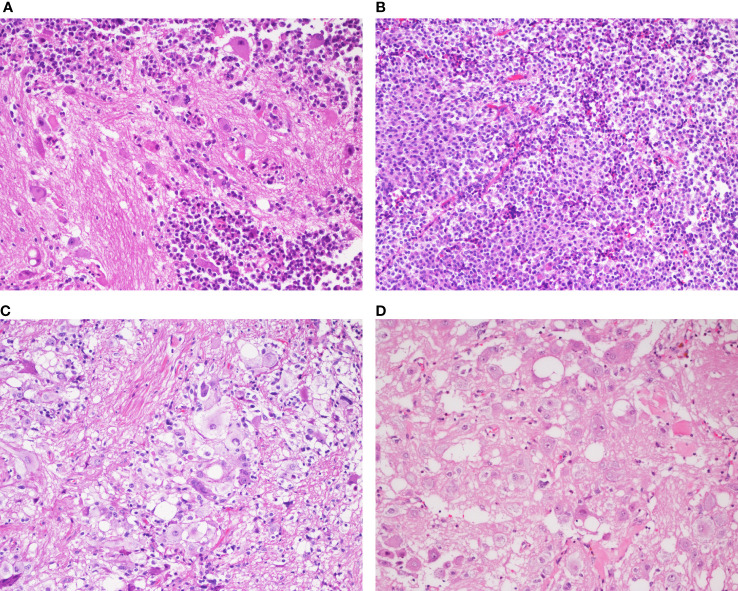
Histopathological analysis of the mixed gangliocytoma-pituitary adenoma in a representative case (Case 3) **(A)** Two distinct mixed neoplastic cell populations are observed. **(B)** The adenomatous component consists of small monomorphic cells with oval nuclei and eosinophilic cytoplasm. **(C)** Binucleated cells and masses of neuropils are detected in the neural component. **(D)** The ganglionic cells are large, immature with abundant cytoplasm and decentralized nuclei containing prominent nucleoli. [**(A–D)**, magnification×200].

Macroscopically, the resected surgical specimens were grayish brown tissue with soft texture, and the size ranged from 1.8 cm×1.5 cm×1.4 cm to 3.0 cm×2.7 cm×2.0 cm. Histologically, four cases were composed of pituitary adenomas admixed with ganglionic cells (**Figure 2A**). The adenomatous component consisted of small uniform cells with acidophilic cytoplasm (**Figure 2B**). The ganglionic component showed polyhedral, occasionally binucleated neurons, with prominent fibrillary neuropils in the stroma (**Figures 2C, D**).

**Figure 3:**
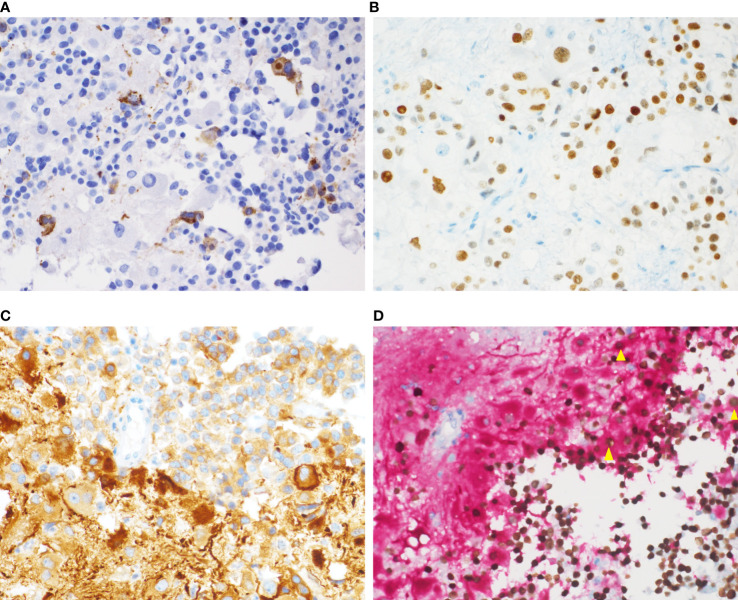
Immunochemical staining of the mixed gangliocytoma-pituitary adenoma. **(A)** Some ganglion-like cells express PRL in the cytoplasm. **(B)** Nuclear PIT1 immunoreactivity is observed in both adenomatous cells and ganglionic cells. **(C)** Fibrillar matrix and large ganglion cells with prominent nucleoli show strong cytoplasmic reactivity for MAP2. **(D)** Double-IHC staining for PIT1 (nuclear; brown) and MAP2 (cytoplasmic; red) shows the coexpression of PIT1 (nuclear) and MAP2 (cytoplasmic) in individual cells (yellow triangle). [**(A–D)**, magnification×400].

**Figure 4:**
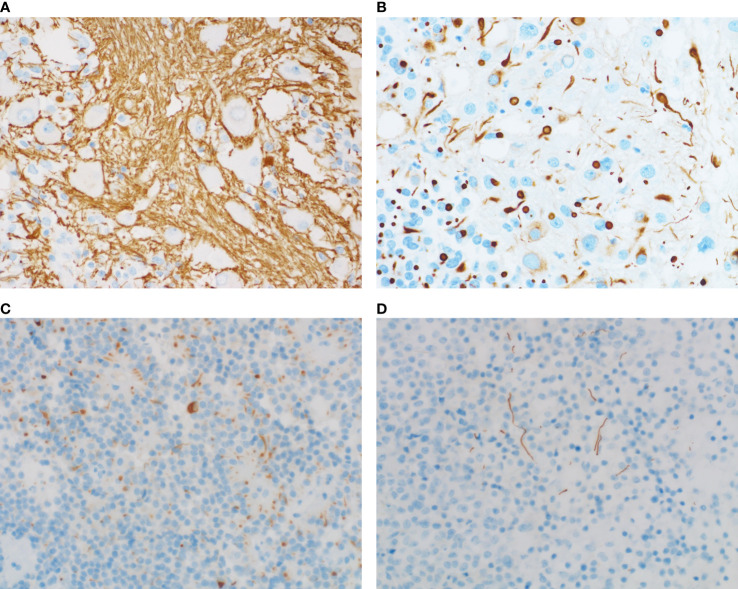
NF staining and CK staining of adenomas with or without neuronal differentiation. **(A)** NF immunostaining shows axotomy-like expression. **(B)** Prominent CK8 staining is noted within fibrous bodies of the ganglion cells. **(C)** NF staining shows a dot-like/cytoplasmic staining pattern. **(D)** Axotomy-like NF coloring can also be seen in adenomas without ganglionic cells. [**(A–D)**, magnification×400].

The expressions of the transcriptional factors were as follows. PIT1 were found to be expressed in all four cases. T-PIT and SF-1 were all negative. In all four cases, the expression of GH, PRL (**Figure 3A**) and PIT1 (**Figure 3B**) was observed in both the adenomatous component and a fraction of the ganglionic component. Large ganglionic cells were positive for microtubule-associated protein 2 (MAP2) (**Figure 3C**). Furthermore, PIT1/MAP2 double-IHC staining was performed. MAP2 and PIT1 were coexpressed in some ganglionic cells (**Figure 3D**). Although adenomatous and ganglionic regions varied in histology, there were different numbers of cells with neuronal differentiation expressing PIT1. Axotomy-like expression was observed in ganglionic cells by NF staining (**Figure 4A**), while typical adenomatous cells did not express NF. Additionally, the axotomy-like appearance of NF staining can be seen in a distant area of adenoma regions. A typical dot-like paranuclear CK8 immunoreactivity pattern was observed in ganglion cells (**Figure 4B**), in addition to expression in the fibrous bodies of the adenomatous cytoplasm. TTF-1, GFAP and CD34 were negative in all four cases. Immunostain for a mutation-specific antibody and mutation testing has shown no BRAF V600E mutation (4 case tested). Ki-67 proliferation index was from 1% to 7%. The immunohistochemical results of pituitary transcription factors, pituitary hormones, neuronal markers and Ki-67 proliferation indexs are summarized in **Table 2**. According to the 2017 World Health Organization Neuroendocrine Tumor Classification Guideline, final pathological diagnoses of four cases were mixed gangliocytoma-adenomas. The adenomatous components were all sparsely granulated mammosomatotroph adenomas.

**Table 2:** List of immunohistochemical results.

	Case1		Case2		Case3		Case4	
	Ad	GC	Ad	GC	Ad	GC	Ad	GC
SF-1	–	–	–	–	–	–	–	–
T-PIT	–	–	–	–	–	–	–	–
PIT1	+	+	+	+	+	+	+	+
PRL	+	+	+	+	+	+	+	+
GH	w+	w+	+	+	+	+	+	+
ACTH	–	–	–	–	–	–	–	–
LH	–	–	–	–	–	–	–	–
TSH	–	–	–	–	–	–	–	–
FSH	–	–	–	–	–	–	–	–
CK8	+	+	+	+	+	–	+	–
Syn	+	+	+	+	+	+	+	+
NeuN	–	–	–	–	–	–	–	–
MAP2	–	+	–	+	–	+	–	+
CR	–	+	–	+	–	+	–	+
NF	–	+	–	+	–	+	–	+
P53	/	/	+	+	+	+	+	+
Ki-67	2%	<1%	7%	<1%	3%	<1%	1%	<1%
TTF-1	–	–	–	–	–	–	–	–
GFAP	–	–	–	–	–	–	–	–
CD34	–	–	–	–	–	–	–	–
BRAF	–	–	–	–	–	–	–	–

A, Adenomatous component; GC, ganglionic cells; w+, weak positive.

A total of 111 cases of adenomas without ganglionic cells were included in this study. The expression of NF was investigated in this series. Accordingly, NF positivity was noted in 7 cases. The incidence of neuronal differentiation in this series was 6%. Among them, 4 cases were prolactinomas, 2 cases were somatotroph adenomas, and 1 case was corticotroph adenoma. 6/7 cases were PIT1-positive adenomas. And the remaining one case is T-PIT-positive adenoma. NF immunostaining showed diverse expression patterns, such as paranuclear, cytoplasmic (**Figure 4C**) and axotomy-like coloring (**Figure 4D**). From these findings, we deduce that neuronal differentiation in adenomas without ganglionic cells may not be a rare phenomenon.

## Discussion

Mixed gangliocytoma adenoma is composed of neoplastic mature ganglion cells in combination with pituitary adenomas accompanied by clinical symptoms caused by hypersecretion. GC/MGA was first reported in 1919 by Greenfield et al. and was named choristoma (3). With a deeper understanding of the disease, more varying terminologies have been used to describe it, including neuronal choristoma, choristoma, adenohypophysial choristoma, ganglioneuroma, and pituitary adenoma with neuronal choristoma [PANCH] (4).

To date, 148 cases of GC/MGA have been reported in the literature (5–35). Most of the cases involved female patients with an average age of 44.5 years. The main clinical manifestations of GC/MGA are acromegaly and lactational amenorrhea syndrome, while a few cases present as Cushing´s syndrome or hyperprolactinemia. Patients with acromegaly presented with coarse facial features and acral enlargement. A diagnosis of acromegaly is confirmed biochemically by detection of increased serum IGF-1 concentrations and high serum levels of GH that are not suppressed in an OGTT. Random GH level <1.0µg/L associated with a normal IGF-1 level represents the therapeutic goal and correlates with optimal disease control. A nadir GH level <1 µg/L after OGTT is associated with improved long-term outcomes and lower mortality risk in patients after surgery. Of the 4 MGA patients in this study, 3/4 patients suffered acromegaly, 1/2 patients exhibited headaches, and 1/4 patient experienced amenorrhea, in accordance with the literature. GH serum level was elevated in all cases but Case 2. Nadir GH level after OGTT was >1.0µg/L in Case 2. After 3 months of follow-up, the serum GH levels returned to the normal range in all patients.

Concomitant pituitary adenomas demonstrated by immunohistochemistry in MGAs include somatotroph adenoma, corticotroph adenoma, lactotroph adenoma, mammosomatotroph adenoma and thyrotroph adenoma. Furthermore, GH, PRL, and corticotropin-releasing hormone (CRH) are usually positive in the majority of cases. Radiographically, there was no significant difference in imaging examination between MGA and pituitary adenomas. The lesion was hypointense on the T1-weighted image without enhancement of the mass and hyperintense on the T2-weighted image (15).

Histologically, MGA is composed of two distinct neoplastic cell populations with no clear boundaries. One is a cluster of ganglion cells, and the other is pituitary adenoma. The gangliocytic component consists of irregularly oriented cells with eccentric nuclei containing prominent nucleoli and basophilic cytoplasm. The adenoma component consists of small monomorphic cells with round to ovoid nuclei, delicately stippled chromatin and moderately abundant cytoplasm. A preponderance of pituitary adenoma is sparsely granulated somatotroph adenoma.

Differential diagnosis may mainly concern ganglioglioma. Ganglioglioma is composed of neoplastic mature ganglion cells in combination with neoplastic glial cells. GC/MGA is devoid of neoplastic glial cells. GFAP may aid the differential diagnosis. Meanwhile, CD34 is consistently expressed in 70-80% and BRAF V600E mutation occur in 20-60% of investigated cases of gangliogliomas. The detection of CD34 and BRAF V600E may also be useful for the differential diagnosis.

The histogenesis of pituitary GC/MGA is currently not clear. There are three main hypotheses about the pathogenesis of these tumors: (1) Excess GHRH produced by primary gangliocytoma stimulates the adenomatous formation (36); (2) Both ganglion cells and adenoma cells might arise from a common stem/progenitor cell (37); (3) The neuronal component originates from neuronal differentiation of a preexisting pituitary adenoma (38).

The theory of neuronal differentiation has received increasing recognition. Neuronal transformation is observed in many neuroendocrine cells *in vitro*, including carcinoid tumors, small cell carcinoma of the lung, pheochromocytoma and insulin-producing pancreatic islet cell tumors (39–41). Ultrastructural analysis has found evidence of intermediate cells between adenomatous and ganglionic cells, characterized by neuronal type RER and immunoreactivity for pituitary hormones and low-molecular weight keratin in the perikarya (38). In the present cases, both adenomatous and ganglionic components expressed PIT1. Immunostaining for PRL showed cytoplasmic positivity, and CK8 immunostaining showed strong dot-like perinuclear positivity in ganglionic cells. Meanwhile, a few pituitary adenomatous cells had a neuronal phenotype confirmed by the neuron-related marker NF without morphological characterization of neurons. This result supports the possibility that transdifferentiation could be a potential underlying mechanism of mixed pituitary adenoma–gangliocytomas. Due to the limited number of cases, the effect of neuronal components on the prognosis of MGAs is not yet clear.

Surgery constitutes the primary form of treatment for most patients with MGA/GC. Transsphenoidal tumor resection is the procedure of choice. The rate of surgical success is closely associated with the size and degree of invasiveness of the tumor. Results with large tumors are worse and tumors with evidence of invasion have poor long-term results. Medical therapy has an important role in the management of acromegaly, including dopamine agonists (DAs), the GH receptor antagonist pegvisomant (PEG) and the second-generation SRL pasireotide (PAS) (42). The existence of neural components, however, has no influence on aggressiveness and the risk of recurrence after surgical resection (2).

## Data Availability Statement

The raw data supporting the conclusions of this article will be made available by the authors, without undue reservation.

## Ethics Statement

Written informed consent was obtained from the individual(s) for the publication of any potentially identifiable images or data included in this article.

## Author Contributions

LZ and XY: Conceptualization, Methodology, Formal Analysis, Writing - Original Draft. HC, PZ, YC, QZ, LH, MW, GL, and PW: Immunohistochemistry. CJ, JT, and SZ: Supervision, Writing -Review & Editing. XW: Conceptualization, Funding Acquisition, Resources, Supervision, Writing - Review and Editing. All authors contributed to the article and approved the submitted version.

## Funding

This work was supported in part by Fujian Medical University Startup Fund for scientific research (2020QH1053), in part by National Natural Science Foundation of China (81900767), Natural Science Foundation of Fujian Province (2018J01155).

## Conflict of Interest

The authors declare that the research was conducted in the absence of any commercial or financial relationships that could be construed as a potential conflict of interest.

## Publisher’s Note

All claims expressed in this article are solely those of the authors and do not necessarily represent those of their affiliated organizations, or those of the publisher, the editors and the reviewers. Any product that may be evaluated in this article, or claim that may be made by its manufacturer, is not guaranteed or endorsed by the publisher.

## References

[1] Lopes MBS. The 2017 World Health Organization Classification of Tumors of the Pituitary Gland: A Summary. Acta Neuropathologica (2017) 134(4):521–35. doi: 10.1007/s00401-017-1769-8 28821944

[2] Cossu G Daniel RT Messerer M. Gangliocytomas of the Sellar Region: A Challenging Diagnosis. Clin Neurol neurosurg (2016) 149:122–35. doi: 10.1016/j.clineuro.2016.08.002 27521460

[3] Greenfield JG. The Pathological Examination of Forty Intracranial Neoplasms. Brain (1919) 42(1):29–85. doi: 10.1093/brain/42.1.29

[4] Lloyd RV OR Kloppel G Rosai J. WHO Classification of Tumours of the Endocrine Organs. 4th edn. Lyon: International Agency for Research on Cancer (2017) p. 48–9.

[5] Balci S Saglam A Oruckaptan H Erbas T Soylemezoglu F. Pituitary Adenoma With Gangliocytic Component: Report of 5 Cases With Focus on Immunoprofile of Gangliocytic Component. Pituitary (2015) 18(1):23–30. doi: 10.1007/s11102-013-0551-8 24430434

[6] Domingue ME Marbaix E Do Rego JL Col V Raftopoulos C Duprez T. Infrasellar Pituitary Gangliocytoma Causing Cushing's Syndrome. Pituitary (2015) 18(5):738–44. doi: 10.1007/s11102-014-0595-4 25183169

[7] Petrakakis I Pirayesh A Krauss JK Raab P Hartmann C Nakamura M. The Sellar and Suprasellar Region: A "Hideaway" of Rare Lesions. Clinical Aspects, Imaging Findings, Surgical Outcome and Comparative Analysis. Clin Neurol Neurosurg (2016) 149:154–65. doi: 10.1016/j.clineuro.2016.08.011 27540757

[8] Donadille B Villa C Gaillard S Christin-Maitre S. Gangliocytoma: Outcome of a Rare Silent Pituitary Tumour. BMJ Case Rep (2017) 2017. doi: 10.1136/bcr-2016-218859 PMC5337629 28232376

[9] Teramoto S Tange Y Ishii H Goto H Ogino I Arai H. Mixed Gangliocytoma-Pituitary Adenoma Containing GH and GHRH Co-Secreting Adenoma Cells. Endocrinol Diabetes Metab Case Rep (2019) 2019. doi: 10.1530/EDM-19-0099 PMC6790896 31581122

[10] Robertson DM Hetherington RF. A Case Of Ganglioneuroma Arising In The Pituitary Fossa. J Neurol Neurosurg Psychiatry (1964) 27(3):268–72. doi: 10.1136/jnnp.27.3.268 PMC495739 14175298

[11] Asa SL Kovacs K Tindall GT Barrow DL Horvath E Vecsei P. Cushing's Disease Associated With an Intrasellar Gangliocytoma Producing Corticotrophin-Releasing Factor. Ann Internal Med (1984) 101(6):789–93. doi: 10.7326/0003-4819-101-6-789 6333843

[12] McCowen KC Glickman JN Black PM Zervas NT Lidov HG Garber JR. Gangliocytoma Masquerading as a Prolactinoma. Case Report. J neurosurg (1999) 91(3):490–5. doi: 10.3171/jns.1999.91.3.0490 10470826

[13] Geddes JF Jansen GH Robinson SF Gömöri E Holton JL Monson JP. 'Gangliocytomas' of the Pituitary: A Heterogeneous Group of Lesions With Differing Histogenesis. Am J Surg Pathol (2000) 24(4):607–13. doi: 10.1097/00000478-200004000-00017 10757410

[14] Isidro ML Iglesias Díaz P Matías-Guiu X Cordido F. Acromegaly Due to a Growth Hormone-Releasing Hormone-Secreting Intracranial Gangliocytoma. J Endocrinol Invest (2005) 28(2):162–5. doi: 10.1007/BF03345360 15887863

[15] Qiao N Ye Z Wang Y Li S Mao Y Bao W. Gangliocytomas in the Sellar Region. Clin Neurol neurosurg (2014) 126:156–61. doi: 10.1016/j.clineuro.2014.08.034 25259876

[16] Levitus CF Charitou MM. AN INCIDENTAL COLLISION TUMOR OF THE SELLA TURCICA. AACE Clin Case Rep (2019) 5(4):e247–9. doi: 10.4158/ACCR-2019-0013 PMC6873830 31967045

[17] He M Zheng N Zhang J Hu Z You G Ren Q. Growth Hormone-Secreting Adenoma Coexisted With Gangliocytoma: A Rare Case. Int J Clin Exp Pathol (2018) 11(7):3785–8. PMC6962879 31949764

[18] Novello M Gessi M Doglietto F Anile C Lauriola L Coli A. Characteristics of Ganglion Cells in Pituitary Gangliocytomas. Neuropathol Off J Japanese Soc Neuropathol (2017) 37(1):64–8. doi: 10.1111/neup.12322 27400662

[19] Lopes MB Sloan E Polder J. Mixed Gangliocytoma-Pituitary Adenoma: Insights on the Pathogenesis of a Rare Sellar Tumor. Am J Surg Pathol (2017) 41(5):586–95. doi: 10.1097/PAS.0000000000000806 28079576

[20] Yano S Hide T Uekawa K Honda Y Mikami Y Kuratsu JI. Mixed Pituitary Gangliocytoma and Prolactinoma Resistant to the Cabergoline Treatment. World neurosurg (2016) 95:620.e617–620.e622. doi: 10.1016/j.wneu.2016.08.011 27535625

[21] Jukes A Allan R Rawson R Buckland ME. Growth Hormone Secreting Pituitary Adenoma With Admixed Gangliocytoma and Ganglioglioma. J Clin Neurosci Off J Neurosurg Soc Australasia (2016) 31:202–4. doi: 10.1016/j.jocn.2016.02.024 27068013

[22] Angelstein I. Pathogenesis of Acromegaly. Deutsche Z fur Nervenheilkunde (1953) 170(4):337–48. doi: 10.1007/BF00242976 13116789

[23] Muller W Marcos F. [The Occurrence of Ganglion Cells in a Pituitary Tumor]. Virchows Archiv fur pathologische Anatomie und Physiologie und fur klinische Med (1954) 325(6):733–6. doi: 10.1007/BF00955104 13226715

[24] Jakumeit HD Zimmermann V Guiot G. Intrasellar Gangliocytomas. Report of Four Cases. J neurosurg (1974) 40(5):626–30. doi: 10.3171/jns.1974.40.5.0626 4817807

[25] Asa SL Bilbao JM Kovacs K Linfoot JA. Hypothalamic Neuronal Hamartoma Associated With Pituitary Growth Hormone Cell Adenoma and Acromegaly. Acta Neuropathologica (1980) 52(3):231–4. doi: 10.1007/BF00705811 7445985

[26] Rhodes RH Dusseau JJ Boyd AS Jr. Knigge KM. Intrasellar Neural-Adenohypophyseal Choristoma. A Morphological and Immunocytochemical Study. J neuropathol Exp Neurol (1982) 41(3):267–80. doi: 10.1097/00005072-198205000-00003 7042919

[27] Burchiel KJ Shaw CM Kelly WA. A Mixed Functional Microadenoma and Ganglioneuroma of the Pituitary Fossa. Case Report. J Neurosurg (1983) 58(3):416–20. doi: 10.3171/jns.1983.58.3.0416 6827330

[28] Fischer EG Morris JH Kettyle WM. Intrasellar Gangliocytoma and Syndromes of Pituitary Hypersecretion. Case Report. J Neurosurg (1983) 59(6):1071–5. doi: 10.3171/jns.1983.59.6.1071 6631503

[29] Asa SL Scheithauer BW Bilbao JM Horvath E Ryan N Kovacs K. A Case for Hypothalamic Acromegaly: A Clinicopathological Study of Six Patients With Hypothalamic Gangliocytomas Producing Growth Hormone-Releasing Factor. J Clin Endocrinol Metab (1984) 58(5):796–803. doi: 10.1210/jcem-58-5-796 6423659

[30] Bevan JS Asa SL Rossi ML Esiri MM Adams CB Burke CW. Intrasellar Gangliocytoma Containing Gastrin and Growth Hormone-Releasing Hormone Associated With a Growth Hormone-Secreting Pituitary Adenoma. Clin Endocrinol (1989) 30(3):213–24. doi: 10.1111/j.1365-2265.1989.tb02229.x 2512034

[31] Kamel OW Horoupian DS Silverberg GD. Mixed Gangliocytoma-Adenoma: A Distinct Neuroendocrine Tumor of the Pituitary Fossa. Hum Pathol (1989) 20(12):1198–203. doi: 10.1016/S0046-8177(89)80012-7 2591950

[32] Li JY Racadot O Kujas M Kouadri M Peillon F Racadot J. Immunocytochemistry of Four Mixed Pituitary Adenomas and Intrasellar Gangliocytomas Associated With Different Clinical Syndromes: Acromegaly, Amenorrhea-Galactorrhea, Cushing's Disease and Isolated Tumoral Syndrome. Acta Neuropathologica (1989) 77(3):320–8. doi: 10.1007/BF00687585 2922994

[33] Asada H Otani M Furuhata S Inoue H Toya S Ogawa Y. Mixed Pituitary Adenoma and Gangliocytoma Associated With Acromegaly–Case Report. Neurologia Medico-Chirurgica (1990) 30(8):628–32. doi: 10.2176/nmc.30.628 1703643

[34] Slowik F Fazekas I Bálint K Gazsó L Pásztor E Czirják S. Intrasellar Hamartoma Associated With Pituitary Adenoma. Acta neuropathologica (1990) 80(3):328–33. doi: 10.1007/BF00294652 2399812

[35] Saeger W Puchner MJ Lüdecke DK. Combined Sellar Gangliocytoma and Pituitary Adenoma in Acromegaly or Cushing's Disease. A Report of 3 Cases. Virchows Archiv an Int J Pathol (1994) 425(1):93–9. doi: 10.1007/BF00193956 7921420

[36] Kurosaki M Saeger W Lüdecke DK. Intrasellar Gangliocytomas Associated With Acromegaly. Brain Tumor Pathol (2002) 19(2):63–7. doi: 10.1007/BF02478929 12622135

[37] Kontogeorgos G Mourouti G Kyrodimou E Liapi-Avgeri G Parasi E. Ganglion Cell Containing Pituitary Adenomas: Signs of Neuronal Differentiation in Adenoma Cells. Acta Neuropathologica (2006) 112(1):21–8. doi: 10.1007/s00401-006-0055-y 16699777

[38] Horvath E Kovacs K Scheithauer BW Lloyd RV Smyth HS. Pituitary Adenoma With Neuronal Choristoma (PANCH): Composite Lesion or Lineage Infidelity? Ultrastructural Pathol (1994) 18(6):565–74. doi: 10.3109/01913129409021900 7855931

[39] Lach B Rippstein P Benott BG Staines W. Differentiating Neuroblastoma of Pituitary Gland: Neuroblastic Transformation of Epithelial Adenoma Cells. Case Report. J Neurosurg (1996) 85(5):953–60. doi: 10.3171/jns.1996.85.5.0953 8893739

[40] Chen J Hersmus N Van Duppen V Caesens P Denef C Vankelecom H. The Adult Pituitary Contains a Cell Population Displaying Stem/Progenitor Cell and Early Embryonic Characteristics. Endocrinology (2005) 146(9):3985–98. doi: 10.1210/en.2005-0185 15932930

[41] Taniguchi Y Yasutaka S Kominami R Shinohara H. Proliferation and Differentiation of Rat Anterior Pituitary Cells. Anat Embryol (2002) 206(1-2):1–11. doi: 10.1007/s00429-002-0271-8 12478362

[42] Colao A Grasso LFS Giustina A Melmed S Chanson P Pereira AM. Acromegaly. Nat Rev Dis Primers (2019) 5(1):20. doi: 10.1038/s41572-019-0071-6 30899019

